# Systolic T1 mapping for estimation of myocardial diffuse fibrosis

**DOI:** 10.1186/1532-429X-18-S1-Q52

**Published:** 2016-01-27

**Authors:** Vassilis Vassiliou, Sri Anita, Tamir Malley, Claire E Raphael, Upasana Tayal, Aamir Ali, Joban Sehmi, Hasan Bilal, George L Mathew, Gillian C Smith, Karen Symmonds, Andreas Greiser, Bruce S Spottiswoode, Francisco Alpendurada, Dominique Auger, Dudley J Pennell, Peter Gatehouse, Sanjay Prasad

**Affiliations:** 1 CMR, Royal Brompton Hospital, London, United Kingdom; 2 National Heart and Lung Institute, Imperial College London, London, United Kingdom; 3 Medicine, Imperial College London, London, United Kingdom; 4 Siemens Healthcare, Erlangen, Germany; 5 Siemens Healthcare, Chigaco, IL USA

## Background

Parametric T1 mapping currently allows non-invasive estimation of diffuse left-ventricular fibrosis. Imaging for T1 mapping is usually acquired during the diastolic phase. However, in tachycardia and arrhythmia, diastasis is short and imaging challenging. Conversely, systolic T1 mapping might offer an advantage and further enable more accurate ROI delineation for T1 maps as the myocardium is thicker. Although motion is also more likely during image generation. Recent studies using various T1 mapping sequences in systole have usually shown small differences (~1-2%) between systolic and diastolic T1 values [1–4] but older studies had shown larger differences [5]. We investigated the difference between systolic and diastolic T1 mapping using Siemens investigational prototype 448B.

## Methods

10 healthy volunteers (average age 29 ± 6 yrs, 5 male) underwent CMR on a 1.5T scanner (MAGNETOM Avanto, Siemens Healthcare, Germany). Diastolic native T1 maps were taken after manually identifying diastasis from the short-axis cine (SAX) and calculating the diastolic trigger delay (DTD) for each person. Systolic native T1 mapping was undertaken subsequently by manually identifying peak systole from SAX and calculating the systolic trigger delay (STD). Each patient had 4 native T1 maps (2 basal and 2 mid level) to enable intra-study reproducibility assessment. These values were used to obtain an average basal and a separate average mid-level T1 value as well as an average of all four values to estimate global T1. We also calculated the impact of timing on the native-blood T1 as potentially needed for ECV calculation.

## Results

There was moderate correlation between global diastolic and systolic T1 values (R = 0.68, ICC 0.64) with the mid-slice showing better correlation (table 1). Overall, the global native diastolic T1 values were ~10 ms higher than the systolic values (difference ~ 1%, 95%CI -34 ms to 55 ms, Bland Altman plot figure 2), supporting a small change reported by recent studies [1–4]. However, the maximum difference seen in a patient was 42 ms, representing an up to 4% difference. The sharpness in image quality for diastolic and systolic mapping was assessed visually and was similar. Intra-study reproducibility was strong with both systolic (ICC = 0.79) and diastolic (ICC = 0.82) T1 mapping. Myocardial thickness was higher in systole, by 1.7 mm in the basal and 2.3 mm in mid-slice.

**Figure 1:**
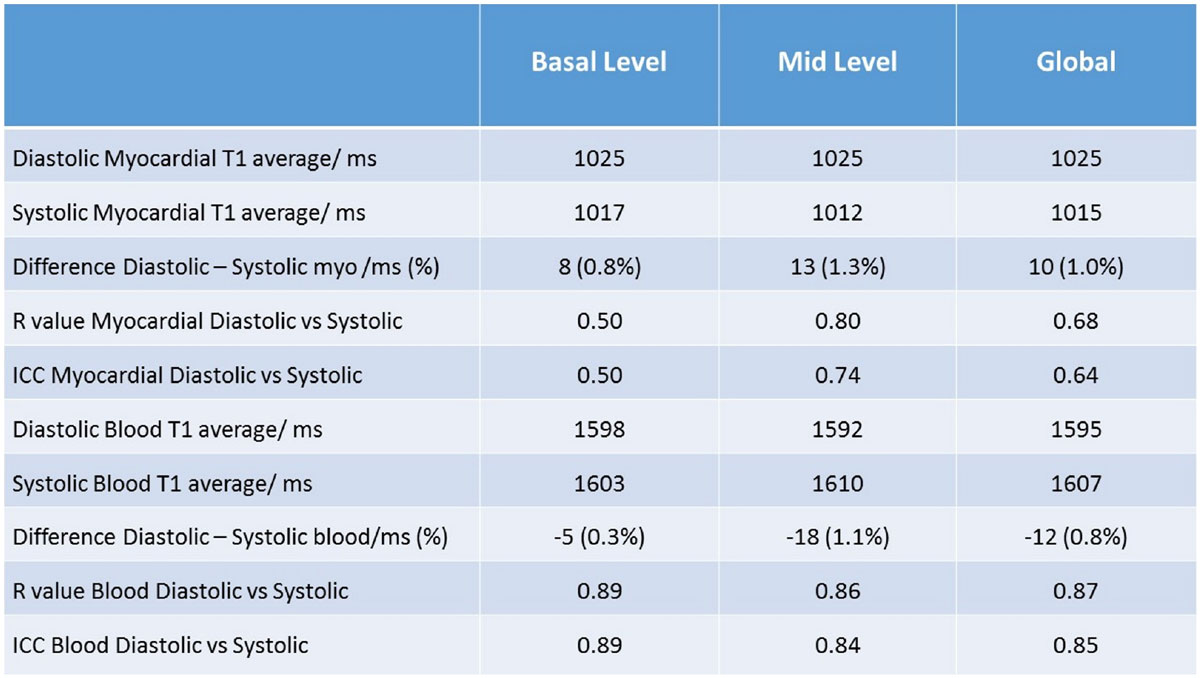
**Table showing the relation between diastolic and systolic native T1 mapping, and the difference between native blood and myocardial T1 values**.

**Figure 2:**
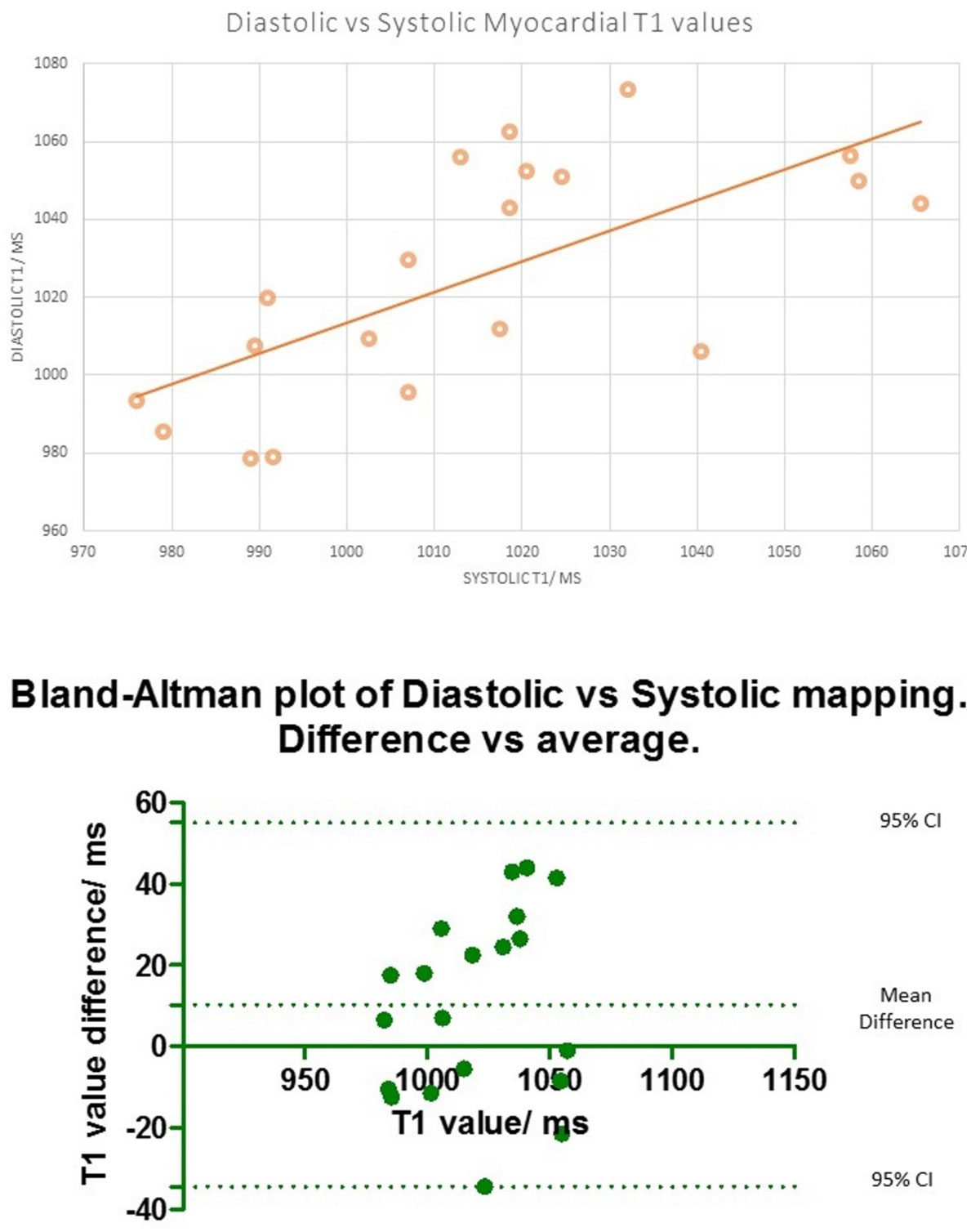
**Top: Correlation between Diastolic and Systolic T1 mapping**. Bottom: Bland Altman plot of difference between diastolic and systolic T1 mapping.The dotted lines represent9th 95% CI.

## Conclusions

Systolic T1 mapping is reproducible in volunteers, with small differences between systole and diastole. This may be of particular interest in patients with arrhythmia, tachycardia or thin myocardium. Further histological validation studies in patients with arrhythmia are indicated, particularly comparing systolic T1 mapping with the arrhythmia-insensitive rapid (AIR) cardiac T1-mapping pulse sequence [6].
